# Novel Functionalized Polythiophene-Coated Fe_3_O_4_ Nanoparticles for Magnetic Solid-Phase Extraction of Phthalates

**DOI:** 10.3390/polym8050117

**Published:** 2016-04-28

**Authors:** Siti Nor Atika Baharin, Norazilawati Muhamad Sarih, Sharifah Mohamad

**Affiliations:** 1 Department of Chemistry, Faculty of Science, University of Malaya, 50603 Kuala Lumpur, Malaysia; atikabaharin@gmail.com (S.N.A.B.); sharifahm@um.edu.my (S.M.); 2 Faculty of Applied Science, Universiti Teknologi MARA, 40450 Shah Alam, Malaysia; 3 University of Malaya Centre for Ionic Liquids, University of Malaya, 50603 Kuala Lumpur, Malaysia

**Keywords:** polythiophene, Fe_3_O_4_ magnetic nanoparticles, phthalates, magnetic solid-phase extraction

## Abstract

Poly(phenyl-(4-(6-thiophen-3-yl-hexyloxy)-benzylidene)-amine) (P3TArH) was successfully synthesized and coated on the surface of Fe_3_O_4_ magnetic nanoparticles (MNPs). The nanocomposites were characterized by Fourier transform infra-red (FTIR), X-ray diffractometry (XRD), Brunauer-Emmett-Teller (BET) surface area analysis, analyzer transmission electron microscopy (TEM) and vibrating sample magnetometry (VSM). P3TArH-coated MNPs (MNP@P3TArH) showed higher capabilities for the extraction of commonly-used phthalates and were optimized for the magnetic-solid phase extraction (MSPE) of environmental samples. Separation and determination of the extracted phthalates, namely dimethyl phthalate (DMP), diethyl phthalate (DEP), dipropyl phthalate (DPP), dibutyl phthalate (DBP), butyl benzyl phthalate (BBP), dicyclohexyl phthalate (DCP), di-ethylhexyl phthalate (DEHP) and di-*n*-octyl phthalate (DNOP), were conducted by a gas chromatography-flame ionization detector (GC-FID). The best working conditions were as follows; sample at pH 7, 30 min extraction time, ethyl acetate as the elution solvent, 500-µL elution solvent volumes, 10 min desorption time, 10-mg adsorbent dosage, 20-mL sample loading volume and 15 g·L^−1^ concentration of NaCl. Under the optimized conditions, the analytical performances were determined with a linear range of 0.1–50 µg·L^−1^ and a limit of detection at 0.08–0.468 µg·L^−1^ for all of the analytes studied. The intra-day (*n* = 7) and inter-day (*n* = 3) relative standard deviations (RSD%) of three replicates were each demonstrated in the range of 3.7–4.9 and 3.0–5.0, respectively. The steadiness and reusability studies suggested that the MNP@P3TArH could be used up to five cycles. The proposed method was executed for the analysis of real water samples, namely commercial bottled mineral water and bottled fresh milk, whereby recoveries in the range of 68%–101% and RSD% lower than 7.7 were attained.

## 1. Introduction

Belonging to non-halogenated esters of phthalic acid, phthalates or phthalate esters are used as plasticizers for nitrocellulose, since it was first recognized in 1880, replacing camphor [1]. Nowadays, phthalates can be found in many different matrices in our environment and are widely utilized in the PVC industries as a plasticizer, from floors, hoses, cables (building materials), toys and medical appliances [2]. Other consumer-based products utilizing phthalates are as a component in inks, adhesive materials, lacquers, sealing and packing materials, materials for treating surfaces, solvents and fixing agents in fragrances, as well as additives in cosmetics [3,4,5]. They become emerging pollutants and harmful to humans, especially children, since they are not chemically bound in plastics and could be leached out into the environment [4]. Thus, the usage of phthalates in the production of toys, baby bottles and pacifiers is banned in many countries [6]. Exposure to phthalates over long-term periods could result in health issues, for example potential carcinogenic effects or critical impact on the hormonal systems, since they own lipophilic properties, which make them easily stored in fatty tissues [7,8,9,10]. The higher molecular weight of phthalates, such as di(2-ethyl-hexyl) phthalate (DEHP), di-*n*-butyl phthalate (DBP) and di-*n*-octyl phthalate (DNOP) often leads to serious health illnesses and is alleged to be carcinogenic and lethal to liver and kidneys, as well as reproductive organs [11,12]. In Malaysia, due to the awareness of the migration of phthalates from food packaging, baby bottles and pacifiers, a regulation has been proposed, which in stated in Food Regulation 27(B) 1985, which regulated plastic materials, and articles shall be examined in agreement with Malaysia Standard MS 2234: “Plastic Materials and Articles Intended to Come into Contact with Food”, which clarified the specific migration limits as follows; 1.5 mg·kg^−1^ for di(2-ethyl-hexyl) phthalate (DEHP), 0.3 mg·kg^−1^ for di-n-butyl phthalate (DBP), 30 mg·kg^−1^ for butyl benzyl phthalate (BBP) and 9.0 mg·kg^−1^ for diisodecyl phthalate (DIDP) [13,14]. Recently, phthalates have been found in polyethylene terephthalate (PET) bottles which may lead to many serious consequences, since the PET bottles are widely used as containers for mineral water, milk and soft drinks. The existence of phthalates in PET bottles may be explained through several possibilities: the type of raw materials, the chemicals or processes involved in bottle manufacturing, the practice of the use of PET bottles, as well as cross contamination in the bottling plant and cap resins [15,16,17,18,19,20]. Studies conducted by Plotan *et al.* and Wagner *et al.* reported that in most of the inspected PET-bottled water samples, endocrine disruptor activity was found [21,22].

Given the unlimited toxic effects arising from these materials, much research has been conducted to find a solution to eliminate its contamination of the environment [23,24]. However, the determination of phthalates in environmental samples is challenging due to their trace amounts and the intervention of an intricate matrix [25]. Therefore, a sample preparation step for the extraction and preconcentration of the analytes is required [26]. Solid phase extraction (SPE) is one of the established and popular methods for sample enrichment prior to analysis using high performance liquid chromatography (HPLC) and gas chromatography (GC) [27,28,29]. The advantages of SPE over liquid-liquid extraction (LLE) are its simplicity, rapidness, that the adsorbent is recyclable, the steadiness, low cost, high enrichment factors and low usage of organic solvents [30]. Numerous types of sorbents were synthesized and used for the determination of plasticizers, for example an octadecyl packed column (C18), α-cyclodextrin functionalized chitosan, poly(styrene–divinylbenzene) polymers and zeolitic imidazolate [31,32,33,34]. The selection of adsorbent plays an important role in SPE, since it can determine the efficiency, anti-interference ability and selectivity of the method for the targeted analytes [35].

Magnetic nanoparticles (MNPs), especially iron oxides, have become one of the most useful materials in numerous applications since their discovery, for example magnetic fluids, catalysis, magnetic resonance imaging and environmental disciplines [36,37,38]. In the application for the removal of pollutants from the environment, the nano-sized particles provide a high surface area to volume ratio, which enhances adsorption capacity and efficiency [39,40]. Moreover, the distinct feature of MNPs is their rapid response to an external magnetic field. This special property, called superparamagnetism, does not preserve magnetism after the elimination of an external field. Thus, it helps to isolate the adsorbents from an aqueous solution in a complex matrix without the need for centrifugation or filtration and can be referred to as magnetic solid-phase extraction (MSPE) [41,42]. Due to the simplicity of the technique, much research has been published on utilizing Fe_3_O_4_ as the adsorbent for MSPE in water samples, for example determining antimicrobial residue, heavy metals, non-steroidal anti-inflammatory drugs and pesticides [43,44,45,46].

**Figure 1:**
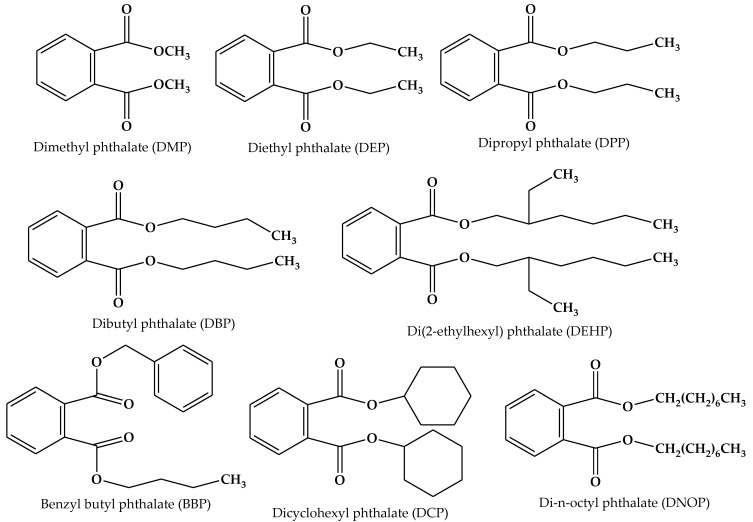
Phthalates used in this study.

However, the smaller the particle size, the more it becomes unstable, which initiates particle accumulation. Moreover, metal oxide may be oxidize easily and reduce its magnetism properties. Therefore, an appropriate surface functionalization can be done, which can be tailored to the specific targeted analyte. The strategy to protect the magnetic core can be either by organic or inorganic compounds, for example Al_2_O_3_, SiO_2_, surfactants, alkyl carboxylates and polymeric coatings [47,48,49,50]. Recently, research articles reported on the utilization of conducting polymers as a coating agent of the MNPs [51,52,53]. These nanocomposites have multifunctional and diverse properties, which may enhance the surface functionalization and protect the magnetic core from environmental agitation. Moreover, it may reduce aggregation and disperse the nanoparticles’ core shell distribution within the suspension media [54]. Herein, we prepared a modified polythiophene containing an additional aromatic ring and aliphatic sides on the surface of Fe_3_O_4_ magnetic nanoparticles (MNPs) to investigate its performance as a magnetic solid-phase extraction of phthalates, as shown in Figure 1. Thus, in this work, the sorbent was further tested for real aqueous samples, including commercial mineral water and commercial fresh milk kept in a PET bottle.

## 2. Materials and Methods

### 2.1. Standard, Reagents and Chemicals

Analytical grade ferric chloride, ferrous chloride, ammonia solution (25 wt %), thiophene, 4-hydroxybenzaldehyde, acetonitrile, potassium permanganate, 4-aminophenol, 3-bromothiophene, 1,6-dibromohexane, *N*-bromosuccinimide, acetic acid, sodium hydrogen bicarbonate, potassium iodide, potassium carbonate, tetrahydrofuran, methanol, hydrochloric acid, acetone and ethyl acetate were purchased from Merck (Darmstadt, Germany). Acetone was procured from Fisher Scientific (Loughborough, UK). Thiophene carboxaldehyde, polyvinyl alcohol and *n*-butyllithium (2.0 M in cyclohexane) were obtained from Sigma Aldrich (Milwaukee, WI, USA). Magnesium sulfate anhydrous, ethanol denatured and hexane were received from J. Kollins (Parkwood, Australia), while dimethyl sulfoxide-d_6_ (DMSO-d_6_) and phthalate esters were purchased from Acros Organics (Geel, Belgium). Ultrapure water was prepared by a model Aqua Max-Ultra ultra-pure water purification system (Zef Scientific Inc., San Diego, CA, USA). Stock solutions of 1000 mg·L^−1^ of standards were prepared by dissolving appropriate amounts of compounds in methanol, which remain stable for three months if stored in a refrigerator at 4 °C. Working standard solutions were prepared daily by diluting the stock standard solution to the required concentrations.

### 2.2. Instruments

The Fourier transform infrared (FTIR) spectra were recorded on a Perkin–Elmer FTIR between 4000 and 400 cm^−1^, with a resolution of 2 cm^−1^. Structural elucidation was determined using ^1^H NMR, JEOL 400 MHz. The pore diameter and surface area of Brunauer-Emmett-Teller (BET) analysis were determined from low-temperature nitrogen adsorption isotherms at 77.40 K using a Quantachrome Autosorb Automated Gas Sorption System (Quantachrome Instruments, Boynton Beach, FL, USA). X-ray powder diffraction (XRD) analysis was conducted with Panalytical model Empyrean (Panalytical, Almelo, Netherlands) at 40 kV and 35 mA using Cu Kα radiation (λ = 1.54059 Å). Morphological analyses of the synthesized products were conducted using transmission electron microscopy (TEM) analysis using an FEI Tecnai G2 spectra microscope (FEI, Hillsboro, OR, USA). The magnetic property was tested using a vibration sample magnetometer (VSM) Model 9600 (Quantum Design Inc., San Diego, CA, USA). Magnetization measurements were carried out in an external field of up to 15 kOe at room temperature.

Separation and detection of target analytes were performed by a Shimadzu 2010 gas chromatograph (Shimadzu, Kyoto, Japan) equipped with a split/splitless injector and a flame ionization detector (FID). A DB-5 Agilent fused-silica capillary column (Agilent, Santa Clara, CA, USA) (30 m × 0.32 mm i.d. × 0.25 µm film thickness) was applied for separation of analytes. Helium (with 99.999% purity) was used as the carrier gas at a constant flow rate of 4 mL·min^−1^. Chromatographic conditions were controlled as described; the temperatures of the injector and detector were set at 260 and 280 °C, respectively. The injection port was operated at splitless mode. Oven temperature was held at 150 °C for 1 min and increased to 280 °C at 8 °C·min^−1^ for 3 min.

### 2.3. Synthesis of Adsorbents

#### 2.3.1. Synthesis of (Phenyl-(4-(6-Thiophen-3-yl-Hexyloxy)-Benzylidene)-Amine) Monomer (**3**) (3TArH)

**Scheme 1:**
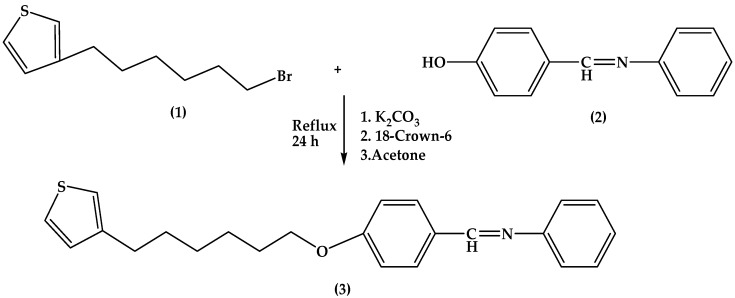
Synthesis pathway for (phenyl-(4-(6-thiophen-3-yl-hexyloxy)-benzylidene)-amine) (3TArH).

Synthesis of (**3**) consists of two steps (Scheme 1). The first step is to prepare the intermediates, which were 3-(6-bromohexyl)thiophene (**1**) and 4-((phenylimino)methyl)phenol (**2**). The second step was combining the two intermediates by the Williamson etherification method [55]. FT-IR spectrums of synthesized compounds were demonstrated in (Figure S1, Supplementary Material)

• 3-(6-bromohexyl)thiophene (**1**): 3-bromothiophene (2 mL, 21.3 mmol) was added to the dry, degassed hexane (50 mL). The reaction started by cooling the flask at −78 °C. *n*-Butyllithium in hexane (10.16 mL) was poured into the reaction flask and stirred for 10 min. THF (5 mL) was injected drop-wise for 15 min and continuously stirred for 1 h, which produced a white precipitate and clear supernatant liquid. The supernatant liquid was removed and changed with hexane/THF (10:1 *v*/*v*, 55 mL). 1,6-dibromohexanes (32.7 mL, 213 mmol) was added and stirred for 2 h. The reaction was stopped with the addition of saturated NaHCO_3_ (50 mL) and diluted diethyl (100 mL). The organic layer was washed with water (100 mL), brine (100 mL), dried with magnesium sulfate anhydrous, treated with decolorizing charcoal, filtered and concentrated in a vacuum to give an oil with an orange color. Excess 1,6-dibromohexane was removed via vacuum distillation (0.04 torr, 55 °C) and purified by silica gel column chromatography (ethyl acetate/hexane, 1/99–5/95 *v*/*v*) to obtain an oily product. Yield: 52%. ^1^H NMR (Figure S2, Supplementary Material) (400 MHz, DMSO-D_6_) δ (ppm): 7.42–6.97, (H*_a, b, c_*), 3.51 (H*_i_*), 2.57 (H*_d_*), 1.6–1.32 (H*_e, f, g, h_*). FTIR (cm^−1^): 3062.45 (C–H aromatic), 2983 and 2912 (C–H (sp^3^)), 1589.22 and 1423.89 (C=C aromatic), 651.02 (C–Br).
• 4-((Phenylimino)methyl)phenol (**2**): 4-hydroxybenzaldehyde (122 mg, 10 mmol) was added to (112 mg, 10 mmol) 2-aminobenzenethiol in 50 mL ethanol. The mixture was refluxed for 3 h. A yellow crystal was obtained after recrystallization with ethanol. Yield: 95%. ^1^H NMR (Figure S3, Supplementary Material) (400 MHz, DMSO-D_6_) δ (ppm): 10.13 (H*_a_*), 8.46 (H*_d_*), 7.80–6.89 (H*_b, c, f_*). FTIR (cm^−1^): 3413.56 (O–H), 3100.34 (C–H aromatic), 1623.05 (C=N) 1589.45 and 1454.65.
• Phenyl-(4-(6-thiophen-3-yl-hexyloxy)-benzylidene)-amine) (**3**): A mixture of 4-((phenylimino)methyl)phenol (1.97 g, 10 mmol), anhydrous potassium carbonate (4.14 g, 30 mmol) and 18-Crown-6 (16.6 mg, 0.1 mmol) was stirred in dried acetone (50 mL) at room temperature. Then, compound 3-(6-bromohexylthiophene) (0.81 g, 2 mmol) was added. The reaction mixture was refluxed under nitrogen with stirring for 24 h. After cooling to room temperature, the reaction mixture was poured into the saturated solution of potassium carbonate. The organic phase was collected and washed by water (3 × 100 mL), dried by anhydrous sodium sulfate and filtered. The solvent was removed by reduced pressure, and the residue was dried by vacuum to produce the crude product. Purification was accomplished by column chromatography on silica with 25% hexane in chloroform to afford the monomer [56]. Yield: 67.6%. ^1^H NMR (Figure S4, Supplementary Material) (400 MHz, DMSO-D_6_) δ (ppm): 8.5 (H*_l_*), 7.8 (H*_k_*), 7.4–6.9 (H*_j, m, n, o_*), 6.6–6.9 (H*_a, b, c_*), 3.97 (H*_i_*), 2.67 (H*_d_*), 1.74–1.41 (H*_e, f, g, h_*). FTIR (cm^−1^): 2938.38, 1617, 1499.9, 1426.71, 1239.71, 1018.26.

#### 2.3.2. Polymerization of 3TArH and Thiophene Monomers on the Surface of MNPs

The preparation of MNP@PTh and MNP@P3TArH NPs involves two steps. Briefly, Fe_3_O_4_ has been prepared by the co-precipitation method [57]. FeCl_3_·6H_2_O (8.48 g, 30 mmol) and FeCl_2_·4H_2_O (2.25 g, 11.3 mmol) were dissolved in 400 mL deionized water under nitrogen atmosphere via vigorous stirring (1000 rpm) at 80 °C. Then, a 20-mL ammonia solution 25% (*w*/*w*) was added to the solution. The color of the bulk solution immediately changed from orange to black. After stirring the mixture for 5 min, the Fe_3_O_4_ NP precipitates were obtained via magnetic decantation and washed three times with deionized water. Finally, the Fe_3_O_4_ NPs were dried in a vacuum oven at 70 °C for 12 h.

The surface of Fe_3_O_4_ NPs was modified by being coated with the newly-designed modified thiophene monomers via oxidation polymerization with the generation of ferric cations on the Fe_3_O_4_ NPs’ surface [54]. Fe_3_O_4_ NPs (1 mmol, 0.235 g) were discrete in polyvinyl alcohol (PVA) aqueous solution (0.001 M). Later, 3TArH (3.64 g, 10 mmol) was added into the mixed solution with vigorous stirring. Subsequently, 30 mL of HCl (0.5 M) solution were introduced into the mixture. Then, the products obtained were dried in a vacuum oven at 70 °C for 12 h. Experiments were repeated using freshly-distilled thiophene monomer (10 mmol, 0.84 g).

### 2.4. Solid Phase Extraction Optimization and Reusability Studies

Factors affecting the extraction efficiency of the proposed method, such as type of adsorbents, pH, extraction time, sample volume, elution solvent, elution solvent volume, desorption time, adsorbent dosage and effect of NaCl, were studied. All of the experiments were performed in triplicate, and the means of the results were used in plotting the optimization curves.

The reusability of the adsorbent was determined with optimized conditions for up to five cycles. The adsorbent was recycled after being washed with methanol and water and dried in vacuum at 70 °C for 12 h.

### 2.5. Analytical Performances and Real Sample Analysis

In order to evaluate the figures of merit of the proposed technique, linearity, the limit of detection (LOD), the limit of quantitation (LOQ) and repeatability were investigated under optimized conditions. The linearity was analyzed through the standard curves ranging from 0.1–50 μg·L^−1^ by diluting appropriate amounts of phthalates stock solution (1000 mg·L^−1^) with methanol and prepared in triplicate. The calibration curves were prepared using 10 spiking levels of analytes. For each level, three replicate experiments were performed.

To evaluate the reliability of the proposed method for the extraction of the plasticizers from real samples, two real samples were selected, spiked and subjected to the MSPE-GC-FID analysis. The two real samples were commercial bottled mineral water and bottled fresh milk.

## 3. Results and Discussion

### 3.1. Characterization of the Samples

**Figure 2:**
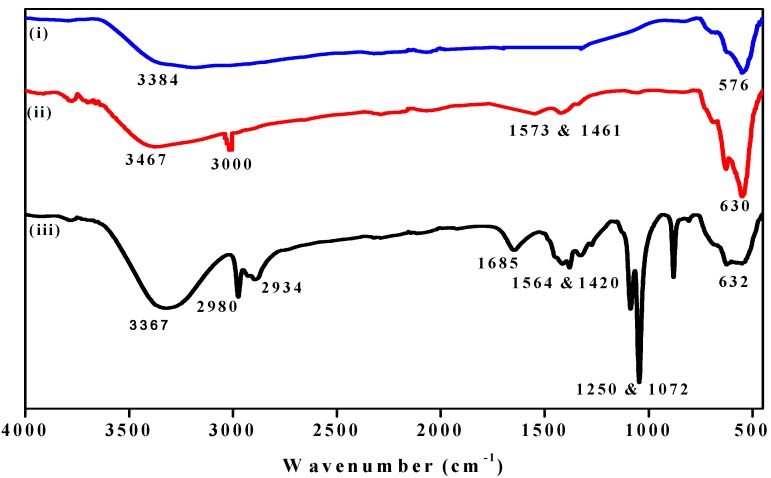
FTIR of: (**i**) MNP; (**ii**) MNP@PTh; (**iii**) MNP@P3TArH.

Figure 2 shows several additional peaks in the spectrum of nanocomposites, proportional to the MNP spectrum, which might be due to the surface functionalization. The strong absorption peaks in the range of ~3400 cm^−1^ for MNP and all nanocomposites indicated the presence of OH vibration, while the peak at 530–632 cm^−1^ corresponds to Fe–O stretching modes [58]. The C–H aromatic stretching peak was observed for all nanocomposites, which falls at 3000 cm^−1^ for MNP@PTh and 2980 cm^−1^ for MNP@P3TArH. C–H sp^3^ stretching (hexyl aliphatic side) occurred at 2934 cm^−1^ for MNP@P3TArH. Schiff base peaks (C=N) were observed at 1674 and 1685 cm^−1^ for MNP@P3TArH [59]. C=C aromatic symmetric and asymmetric absorption bands demonstrated in the range of 1573–1461 cm^−1^ occurred for both nanocomposites. Two absorption band peaks at 1250 and 1072 cm^−1^ indicated the presence of C–O in MNP@P3TArH. Hence, the FTIR study clearly revealed that the MNPs prepared have been successfully functionalized.

**Figure 3:**
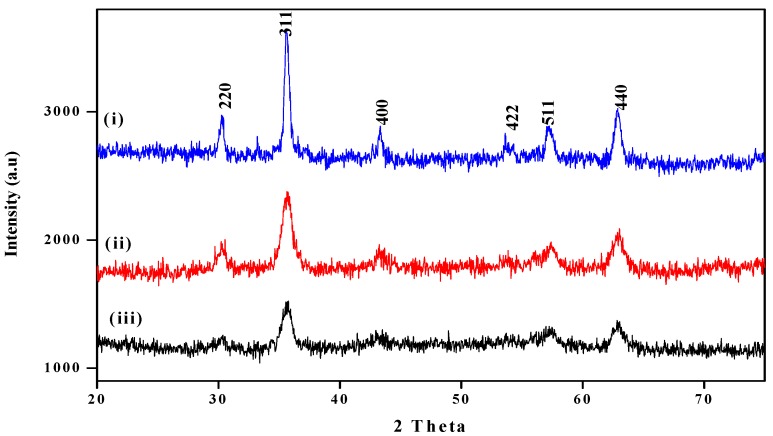
Diffractogram of: (**i**) MNP; (**ii**) MNP@PTh; (**iii**) MNP@P3TArH.

Figure 3 displayed the characteristic peaks observed for the MNPs and all nanocomposites. The peaks of the nanocomposites were slightly wider than unmodified MNP. This may be due to the presence of amorphous and polymeric materials, which coat the surfaces of the MNPs [60]. The characteristic peaks of all nanocomposites were observed at 2θ = 30°, 35.7°, 43°, 53.4°, 57.0° and 62.6°, which are marked by their respective indices ((220), (311), (400), (422), (511) and (440)) [61]. This showed that the surface functionalization does not change the crystalline phase of MNPs [62].

The BET surface area is measured using the multipoint BET method, within the relative pressure (P/P0) range of 0.05–1. As described in (Figure S5, Supplementary Material), the MNPs and all nanocomposites display an H3-type hysteresis loop, based on the Brunauer-Deming-Deming-Teller (BDDT) classification, demonstrating the existence of mesopores with pore diameters between 2 and 50 nm [63]. The pore size and BET surface area of MNPs and nanocomposites are tabulated in Table 1. The reduction in the pore size of nanocomposites is due to the addition of polymers on the surface. Meanwhile, escalation in the surface area could be because of the dispersity of particles that results from the enhancement of the spaces between them [64,65].

**Figure 4:**
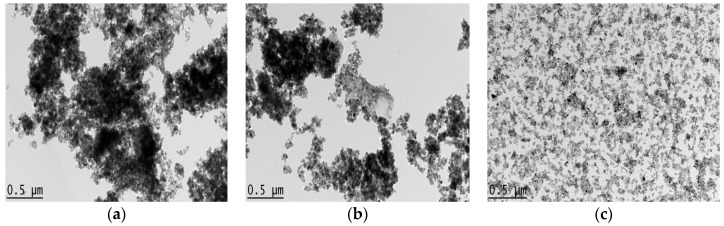
TEM images of: (**a**) MNP; (**b**) MNP@PTh; (**c**) MNP@PTArH.

Morphological analysis of the synthesized products was performed using TEM techniques. As shown in Figure 4, TEM images of all materials demonstrated a sphere-shaped property. From the images, we could clearly observe the good dispersion of the functionalized nanoparticles (MNP@PTArH) in the TEM image. For instance, before polymerization, magnetic nanoparticles were highly agglomerated with each other. After polymerization of MNP with 3TArH, they showed lower agglomeration, and the nanocomposite became well dispersed. The dispersity of the nanocomposite influenced its surface area, as evidence by the BET result of MNP@P3TArH, which is higher compared to MNP@PTh and MNP, as tabulated in Table 1.

The magnetic properties of the samples were recorded at room temperature with an external field of ±15 kOe. Important magnetic variables, such as saturation magnetization (*M*_S_), were evaluated. The maximum saturation (*M*_S_) of MNPs occurred at 69.2 emu·g^−1^, respectively. After surface functionalization, the magnetization of MNP@PTh and MNP@P3TArH was reduced to 65.3 and 61.5 emu·g^−1^ respectively. The magnetization decrease signified the presence of a dead magnetic layer on the surface of the nanocomposites [58]. Although the magnetization has declined, the value is still within the acceptable range, which suggests that it can be applied as the MSPE sorbent [66].

### 3.2. Solid Phase Extraction Optimization and Reusability Studies

#### 3.2.1. Type of Adsorbent

**Figure 5:**
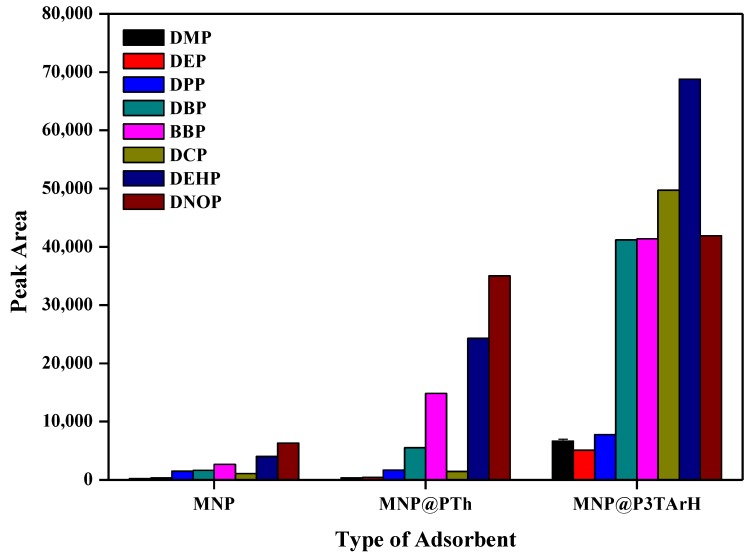
Comparison of MNP@P3TArH with naked MNP and MNP@PTh for the extraction of targeted phthalates.

Hypothetically, the adsorption of phthalates is based on the hydrophobicity and π–π dispersion [67]. To prove that the structure architecture influences the adsorption studies of phthalates, three different types of sorbents, which are naked magnetic nanoparticles (MNP), MNP-PTh and MNP@P3TArH, were tested. As seen in Figure 5, MNP resulted in an insignificant peak area for all of the analytes studied. After the introduction of polythiophene derivatives on the surface of MNP, the peak area of phthalates increased. The presence of aliphatic and aromatic groups in the MNP@P3TArH enhances the dispersion of phthalates, which enhances the π–π dispersion and hydrophobic interaction. As evidenced, butyl benzyl phthalate (BBP) is more prone to the adsorbent with more aromatic sides, as in the MNP@PT3ArH, compared to the other adsorbents. Besides, the high surface area of MNP@P3TArH also contributes to the increase of extraction performance. Since the MNP@P3TArH has demonstrated the high peak area for all analytes studied, it was selected for further MSPE optimization.

#### 3.2.2. Sample pH

**Figure 6:**
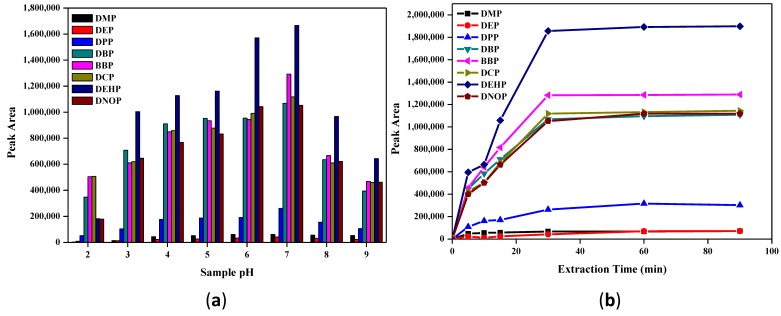
(**a**) Effect of sample pH; (**b**) effect of extraction time for the extraction of targeted phthalates.

To study the influence of the surface charge of adsorbent/adsorbate in the extraction process, experiments were performed under different pH conditions, ranging from pH 2–9. As shown from Figure 6a, the peak areas for phthalates increase when the pH rise from 2–7, but decline later from 8–9. At low pH, C=N, alkoxy in P3TArH was protonated, making the adsorbent surface positively charged. At pH < 7, phthalates hydrolyze to phthalic acid, thus making the carbonyl group nucleophilic, reacting with hydrogen ions in the aqueous solution, producing positive charges. Due to both the absorbate and adsorbent acquiring positive charges, the electrostatic repulsion occurred and retarded the adsorption performance [68]. At basic conditions, the surface adsorbent became negatively charged, while the adsorbate hydrolyzes to phthalate anions, reducing the extraction efficiency [69]. Thus, in neutral pH, the extraction increased due to the absence of electrostatic repulsion that disturbed the extraction capability. As the optimum performance was demonstrated at pH 7, this pH was selected for all of the experiments.

#### 3.2.3. Extraction Time

It has been understood that prolonged extraction time might increase the recovery of analytes. Thus, the influence of extraction time on the recoveries of the analyte has been investigated. As demonstrated in Figure 6b, the peak area increased rapidly for the first 20 min, since more adsorption sites were available and phthalates could easily interact with these sites. After 30 min, the peak area was almost persistent; therefore, 30 min was sufficient to extract the maximum of the target analytes. In order to ensure that the extraction time was satisfactory, further experiments were carried out until 90 min, and they were found to be constant.

#### 3.2.4. Desorption Studies

The elution solvent is one of the crucial parameters to be considered. In order to determine the best elution solvent, the solvent must be able to elute all of the analytes that were retained from the adsorbent in a small volume [70]. Six eluting solvents with dissimilar polarities, namely hexane, toluene, diethyl ether, acetonitrile, methanol and ethyl acetate, were studied.

**Figure 7:**
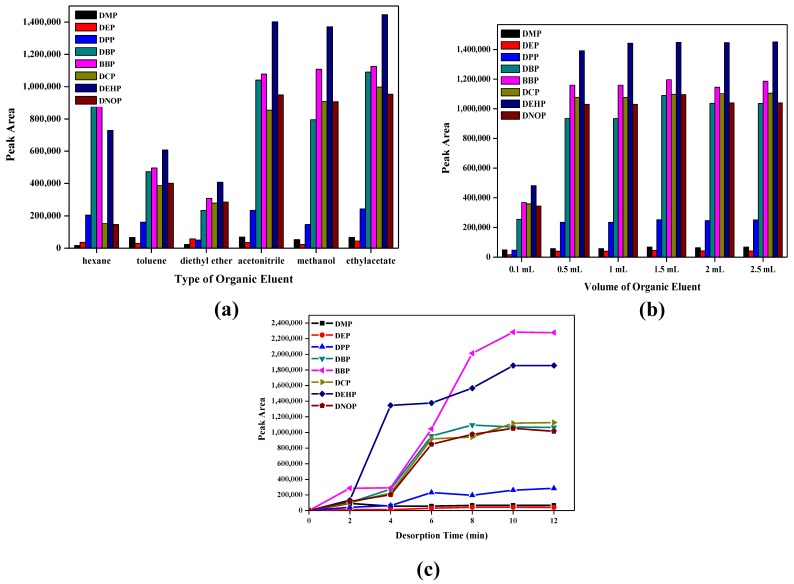
Desorption studies for the extraction of targeted phthalates: (**a**) effect of eluent type; (**b**) volume of organic eluent; (**c**) desorption time.

As evidenced in Figure 7a, polar solvents (acetonitrile, methanol and ethyl acetate) were the best solvents, with high peak areas compared to non-polar solvents (hexane, toluene and diethyl ether), since phthalates contain a polar carbonyl group [71]. Among the polar solvents, ethyl acetate showed high solvent strength, since it gave the maximum peak area for the phthalates studied and was thus selected to be the eluent.

The volume of ethyl acetate was tested from 0.1 mL–2.5 mL. As observed in Figure 7b, the peak area increased from 0.1 mL and remained constant after 0.5 mL. This showed that 0.5 mL may accommodate the maximum phthalates extracted from the sorbent.

Further, desorption time was optimized to investigate the best time taken for the analytes to desorb from the sorbent ranging from 0–12 min. As revealed in Figure 7c, analytes were desorbed rapidly in the first 4 min and started to become linear after 10 min. This indicated that 10 min of time are sufficient to desorb back all of the analytes from the adsorbent. As for the case of BBP, desorption was found to be slower than other phthalates. This could be due to the presence of an additional aromatic ring in BBP, which makes it less polar to the eluent (ethyl acetate). After 6 min of desorption, most of the phthalates had reached near to equilibrium, whereas BBP was desorbed steeply after 6 min until it reached equilibrium at 10 min.

#### 3.2.5. Mass of Adsorbent

**Figure 8:**
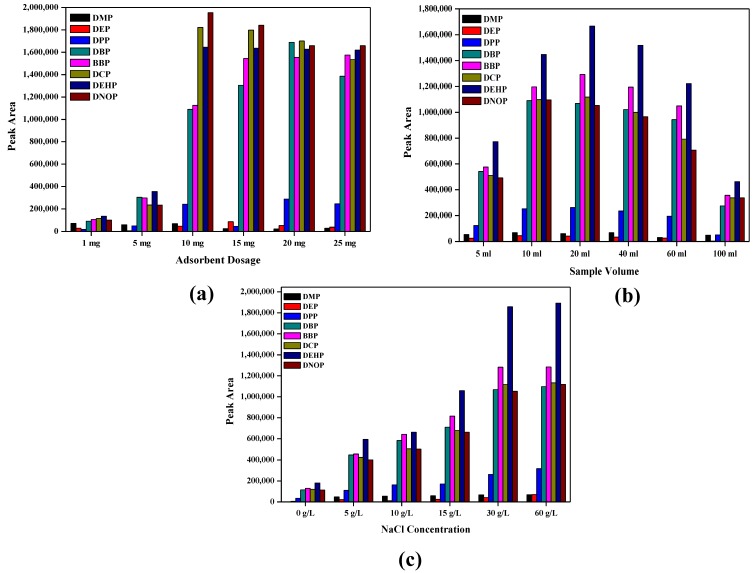
(**a**) Effect of adsorbent dosage; (**b**) effect of sample volume; (**c**) effect of NaCl concentration, for the extraction of targeted phthalates.

Investigation of the adsorbent amount was executed in the range of 1–25 mg. As exposed in Figure 8a, the extraction peak area increased up to 10 mg, but decreased later with a further increase of the adsorbent. Increasing the adsorbent amount provides more active sites for the adsorption of target analytes. However, a high amount of adsorbent at a specific volume has weakened elution efficiency [30]. It is shown that this adsorbent only required a small amount of adsorbent to remove phthalates efficiently, which added the advantage of economic value. Therefore, for further experiments, the adsorbent amount of 10 mg was applied.

#### 3.2.6. Sample Loading Volume

The effect of sample volume was investigated by the extraction of the phthalates ranging from 5–100 mL and shown in Figure 8b. Each sample was spiked with 10 mg·L^−1^ analytes and 10 mg adsorbent. As can be seen, peak area increased until 20 mL and further decreased till 100 mL. A 20-mL volume of sample demonstrated the most efficient extraction. An increase in sample volume could lead to a high distribution of adsorbent to the aqueous phase, which lowered the amount of adsorbent in the volume unit sample solution, and the extraction became less effective [72]. Thus, a 20-mL sample volume was chosen as the optimized sample volume.

#### 3.2.7. Effect of NaCl

Indeed, the addition of salt in the sample matrices effects the extraction efficiency. Thus, studies on the concentration of NaCl ranging from 0–25 g·L^−1^ were conducted. As observed in Figure 8c, peak areas of the studied analytes increased from 0–15 g·L^−1^, but decreased later from 20–25 g·L^−1^. This can be due to the addition of salt, which increases the ionic strength and eventually decreases the solubility of the analytes in the media. However, as the concentration of salt increases, the diffusion rate of the analytes may reduce, since the solvation cage of the analytes is disturbed [51]. Since a 30 g·L^−1^ NaCl concentration gave a high peak area for all analytes studied, it was chosen for subsequent experiments.

#### 3.2.8. Reusability Studies

**Figure 9:**
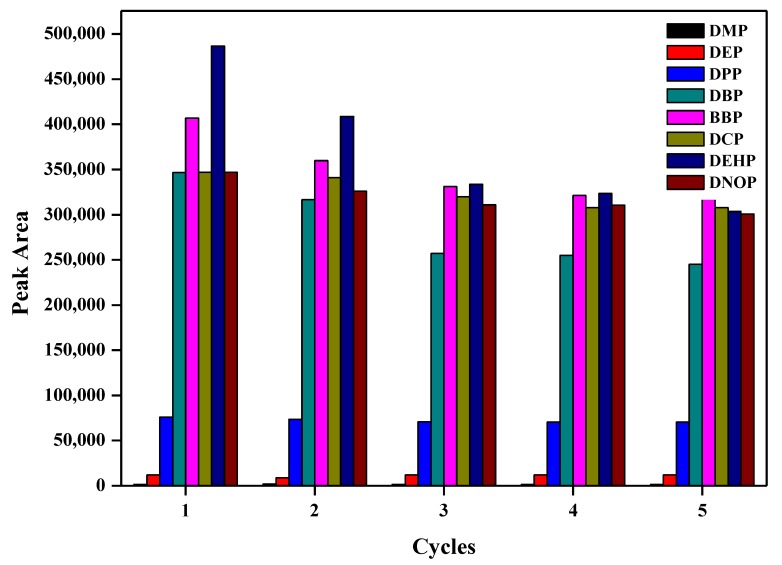
Reusability cycles.

To investigate the probability of reusing and regenerating the sorbent, a reusability test was designed and implemented for Fe_3_O_4_@P3TArH, which was recycled after being washed with methanol and water and was dried in a vacuum at 70 °C for 12 h. From Figure 9, it could be surmised that after five repeated experiments, the adsorbent was still active. This may be due to some of the particles in the adsorbent accumulating due to the heat treatment after several cycles, which decreases the surface area.

### 3.3. Analytical Performances and Real Sample Analysis

The optimized method obtained for the extraction of phthalates using MNP@P3TARH involved the sample at pH 7, 30 min extraction time, ethyl acetate as the elution solvent, 500-µL elution solvent volumes, 10 min desorption time, 10 mg adsorbent dosage, 20-mL sample loading volume and a 15 g·L^−1^ concentration of NaCl. In order to assess the validation of the proposed method, linearity, the limit of detection, the limit of quantitation and repeatability were performed under optimum conditions. Analytical performance figures of merits are tabulated in Table 2.

Calibration curves obtained for the studied phthalates were linear over the range of 0.1–50 µg·L^−1^ with *R^2^* more than 0.99. As per the U.S. EPA standard, the screening of phthalates in drinking water must be done at a concentration above 0.6 µg·L^−1^ [4]. However, the LOD of our method lies within the range of 0.080–0.468, indicating the suitability of this method as an efficient phthalate detector.

Repeatability studies were conducted for inter-day (three consecutives replicates for three days) and intra-day (seven consecutives replicates on the same day). The results were expressed as relative standard deviations (RSD%). This method demonstrated good precision, since the RSD (%) values were in the range of 3%–5% [73]. Comparative studies on the analytical performance between the proposed methods with other developed methods are shown in Table 3. Obviously, the extraction of phthalates using MNP@P3TArH provides sensitivity and repeatability.

**Figure 10:**
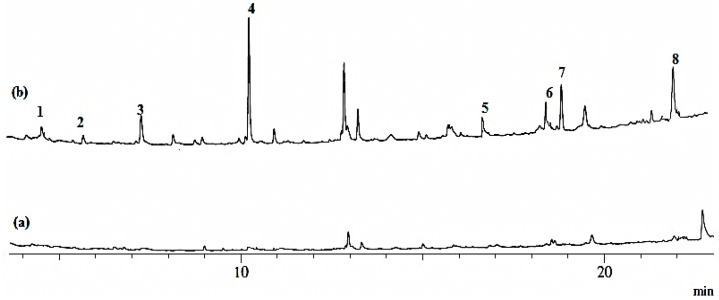
Chromatogram of commercial fresh milk: (**a**) unspiked; (**b**) spiked phthalates (50 µg·L^−1^). Peaks: (1) DMP; (2) DEP; (3) DPP; (4) DBP; (5) BBP; (6) DCP; (7) DEHP; (8) DNOP.

To endorse the reliability of the method using MNP@P3TArH, it was applied to determine phthalates in the water from the mineral water bottle stored at room temperature and commercial fresh milk. Figure 10 shows the chromatogram of commercial fresh milk unspiked and spiked with phthalates. None of the targeted phthalates were found in the water samples under the optimized condition described. To evaluate the matrix effect, all of the samples were spiked with 50 µg·L^−1^ of the phthalates studied. Recoveries and RSD (%) for all of the water samples were determined and are shown in Table 4. From the optimization procedures until the real sample analyses, DMP, DEP and DPP demonstrated lower recoveries; this may be due to the lower molecular weight of phthalates being more prone to aqueous solution than to the adsorbent [76]. From the chromatogram of mineral bottle stored at room temperature as shown in (Figure S6, Supplementary Material), the recoveries obtained for water in the mineral bottle demonstrated higher values compared to the recovery for the milk sample. This might be caused by the matrix effect that holds the analyte in the milk sample to be higher compared to the water sample. RSD (%) values were found to be in the range of 1.3%–5.8%, which indicated a precise method.

## 4. Conclusions

MNP@P3TArH has been successfully synthesized, characterized and utilized as a sorbent for the analysis of GC-FID in the determination of selected phthalates. The optimized conditions of MSPE were carefully selected as follows: sample at pH 7, 30 min extraction time, ethyl acetate as the elution solvent, 500-µL elution solvent volume, 10 min desorption time, 10 mg adsorbent dosage, 20-mL sample loading volume and a 15 g·L^−1^ concentration of NaCl. The steadiness and reusability studies suggested that the MNP@P3TArH could be used up to five cycles without significantly impacting its extraction capacity. The adsorbent covers a wide range of phthalates with a dynamic linear range of 0.1–50 µg·L^−1^ and a limit of detection at 0.08–0.468 µg·L^−1^. The presence of new interfaces (π–π and hydrophobic interactions) among the sorbent and target analytes increased the adsorption capability. The application of MNP@P3TArH as the MSPE sorbent was successfully executed by the analysis of phthalate esters in the mineral water and commercial fresh milk.

## Figures and Tables

**Table 1:** BET pore size and surface area.

Sample	Pore size (nm)	Surface area (m^2^·g^−1^)
MNP	20.2	37.37
MNP@PTh	18.3	95.6
MNP@P3TArH	12.09	103.80

**Table 2:** MNP@P3TArH (magnetic-solid phase extraction (MSPE)) analytical figures of merit.

Analytes	*R^2^*	Linear range (µg·L^−1^)	LOD (µg·L^−1^)	LOQ (µg·L^−1^)	RSD (%) Interday *n* = 3	RSD (%) Intraday *n* = 7
DMP	0.992	0.5–50	0.462	1.539	3.4	4.8
DEP	0.992	0.5–50	0.468	1.562	5.0	4.3
DPP	0.997	0.5–50	0.286	0.954	4.6	3.7
DBP	0.998	0.1–50	0.063	0.213	4.5	4.5
BBP	0.996	0.1–50	0.080	0.268	4.8	4.3
DCP	0.993	0.5–50	0.332	1.106	4.7	4.0
DEHP	0.997	0.1–50	0.054	0.182	3.0	4.0
DNOP	0.997	0.1–50	0.073	0.244	3.6	4.9

**Table 3:** Comparatives study of the proposed method with other MSPE adsorbents for the extraction of phthalates.

Analyte	Method	LOD (µg·L^−1^)	LDR (µg·L^−1^)	RSD (%)	Reference
DBP, DEHP, DOA	MNP@PTh-GC-FID	0.2–0.4	0.4–100	4–12.3	[72]
DPP, DBP, DCP, DNOP	MNP@Chitosan-C18-HPLC-UV	0.012–0.037	0.001–0.01	2.1–6.8	[73]
DBP, DEHP	MNP@Zeolite-GC-FID	2.80–3.2	10–1200	10%–13%	[74]
DMP, DEP, DBP, BBP, DNOP	MNP@ZIF-8-HPLC	0.08–0.24	1–100	<5.5	[75]
DMP, DPP, DEP, DBP, BBP, DCP, DEHP, DNOP	MNP@P3TArH- GC-FID	0.05–0.09	0.1–50	3.0–5.0	This study

**Table 4:** Analysis of analytes in the real samples.

Analyte	MNP@P3TArH MSPE (±RSD%, *n* = 3)	
	Mineral water	Commercial fresh milk
DMP	85(5.8)	68(5.0)
DEP	85 (4.9)	67(3.0)
DPP	88(1.3)	72(7.7)
DBP	95(2.4)	85(3.3)
BBP	93(3.0)	82(3.8)
DCP	90(4.7)	77(5.8)
DEHP	99(1.3)	89(4.5)
DNOP	101(4.2)	91(3.3)

## Supplementary Materials

Supplementary Materials can be found at www.mdpi.com/2073-4360/8/5/117/s1.
