# Supplementary material for: Novel Functionalized Polythiophene-Coated Fe3O4 Nanoparticles for Magnetic Solid-Phase Extraction of Phthalates
Source: Polymers (Basel). 2016 Apr 28;8(5):117. doi: 10.3390/polym8050117 (PMC6431896; doi:10.3390/polym8050117)
Supplement: Supplementary file 1 [file polymers-08-00117-s001.docx]

Novel Functionalized Polythiophene—Coated Fe_3_O_4_ Nanoparticles for Magnetic Solid-Phase Extraction of Phthalates

Siti Nor Atika Baharin, Norazilawati Muhamad Sarih and Sharifah Mohamad

**Figure S1.** FT-IR for compounds (**A**) 4-((phenylimino)methyl)phenol; (**B**) 3-(6-bromohexyl) thiophene and (**C**) (Pheny-(4-(6-thiophen-3-yl-hexyloxy)-benzylidine)-amine).

**
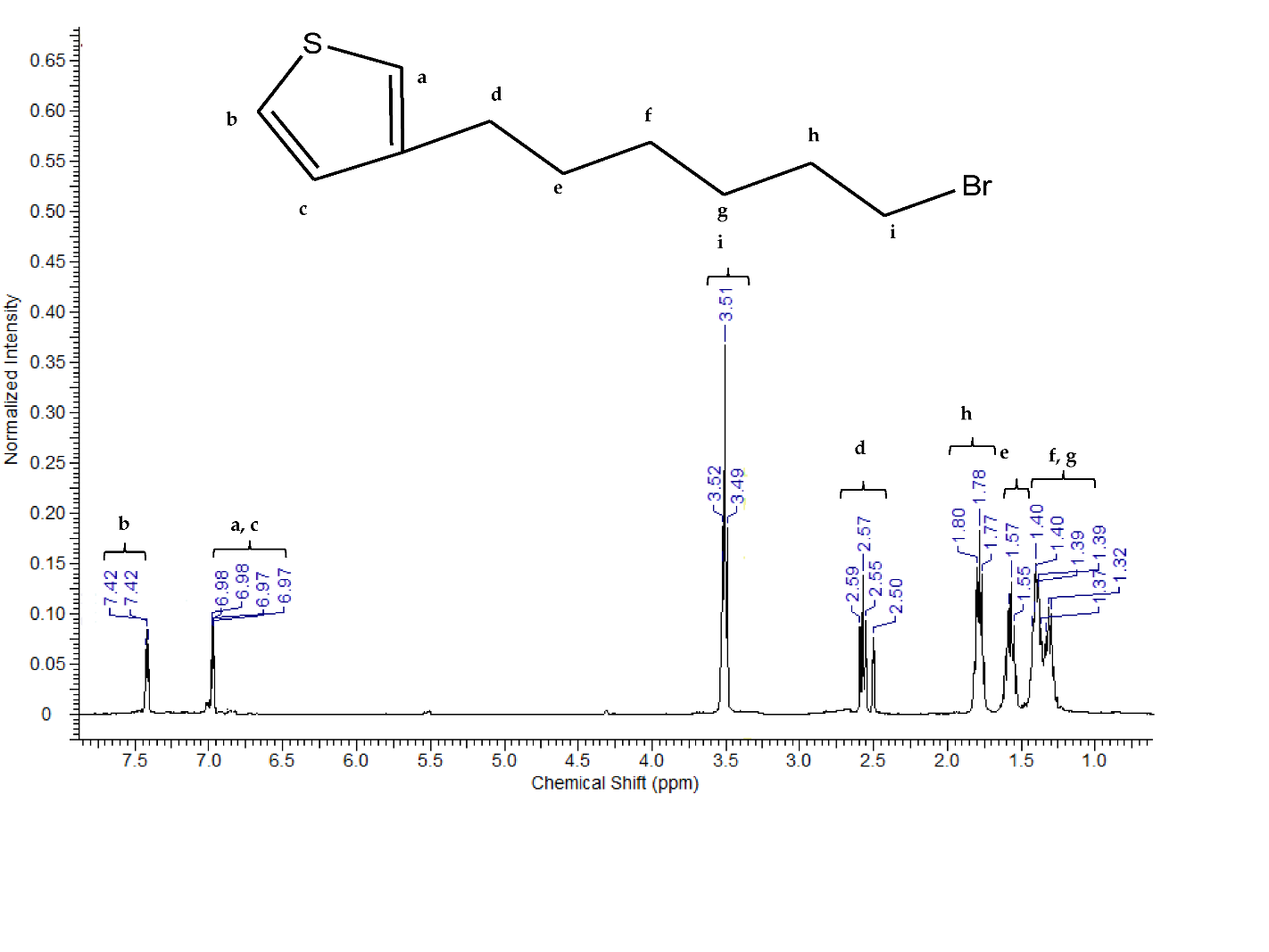
**

**Figure S2.** ^1^H NMR for 3-(6-bromohexyl) thiophene (**1**).


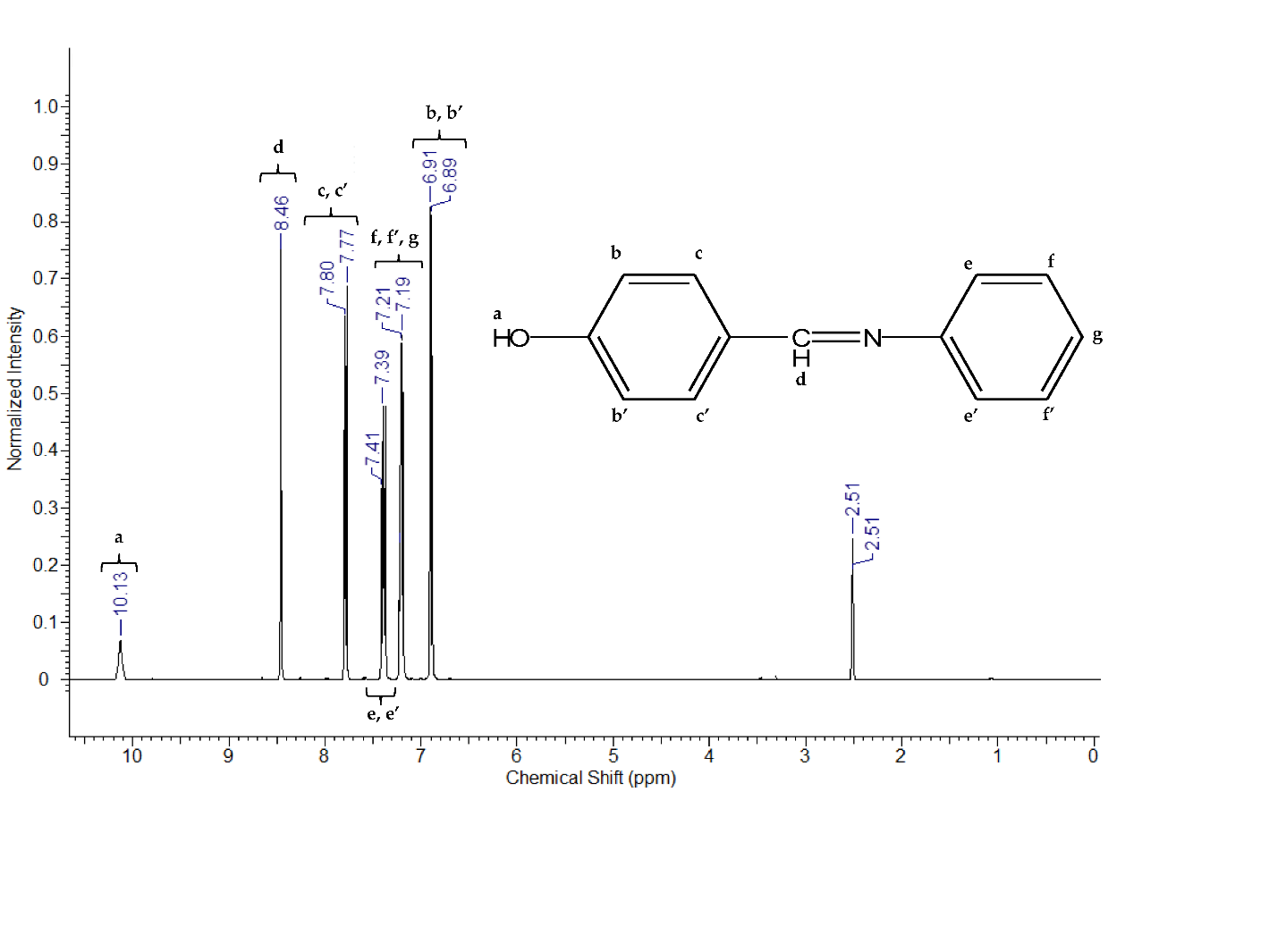


**Figure S3.** ^1^H NMR for 4-((phenylimino)methyl)phenol (**2**).

**
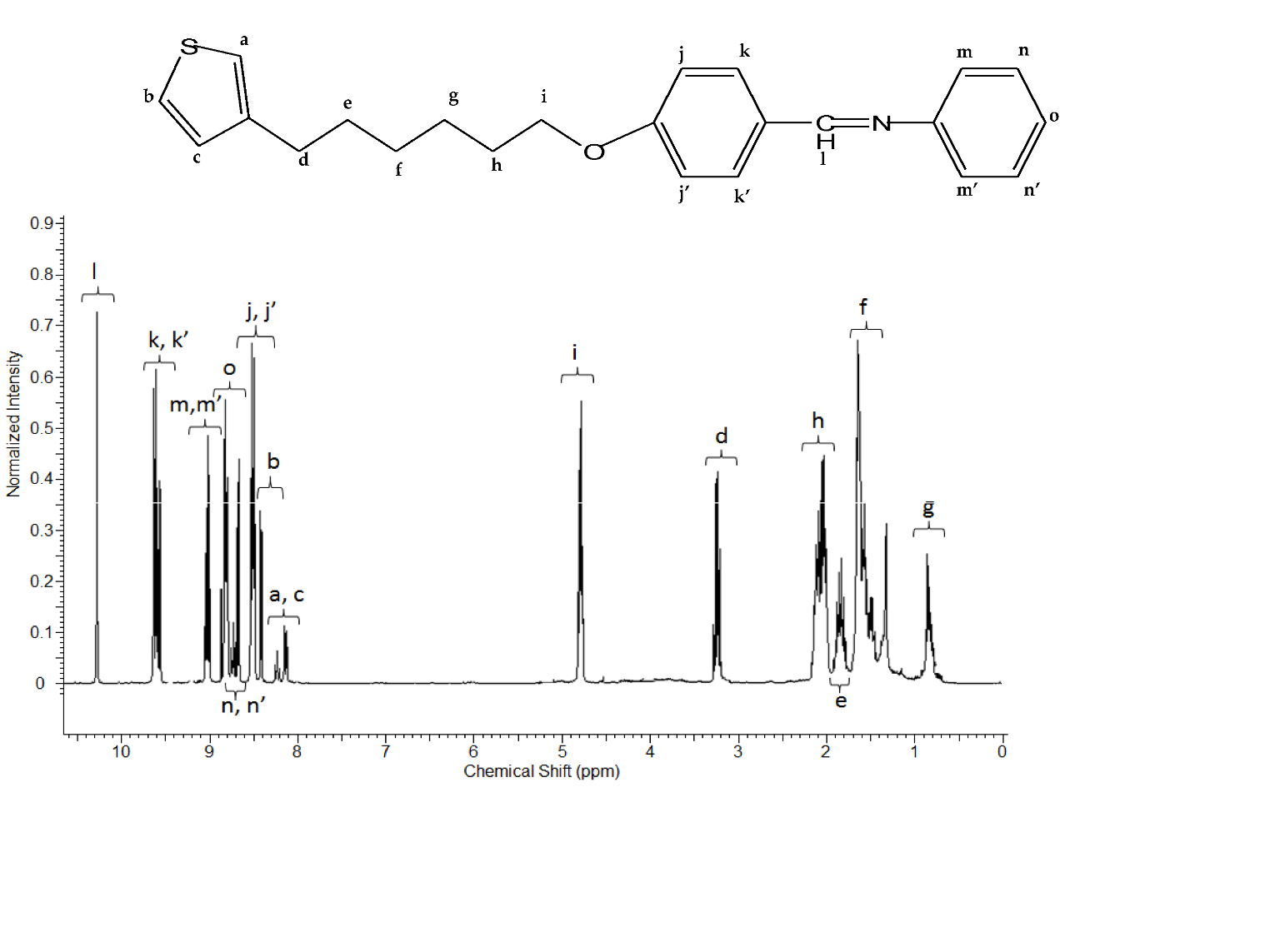
**

**Figure S4.**^1^H NMR for (Pheny-[4-(6-thiophen-3-yl-hexyloxy)-benzylidine]-amine) (**3**).

**
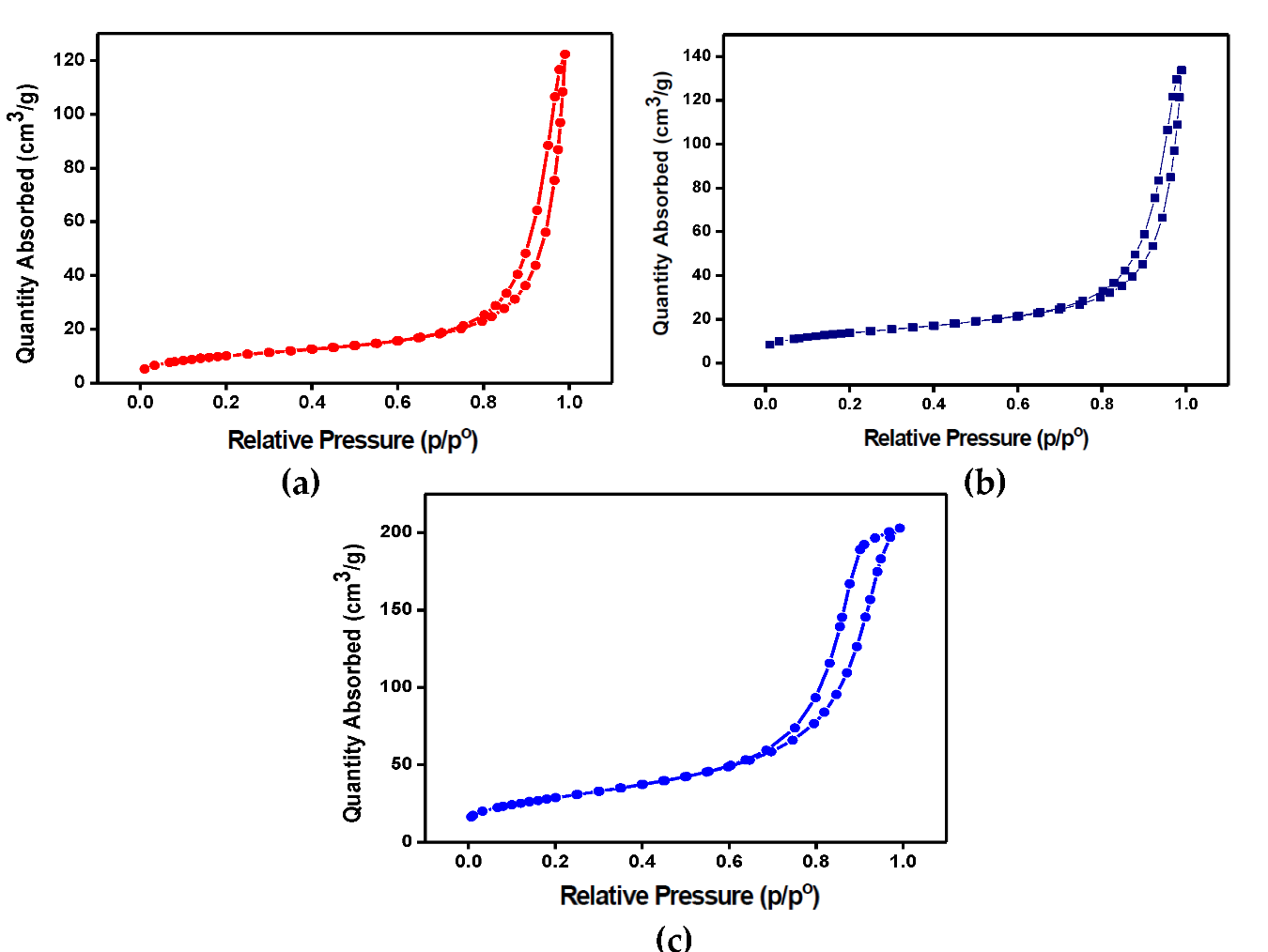
**

**Figure S5.** BET profiles (**a**) MNP; (**b**)MNP@PTh; (**c**) MNP@P3TArH.

**
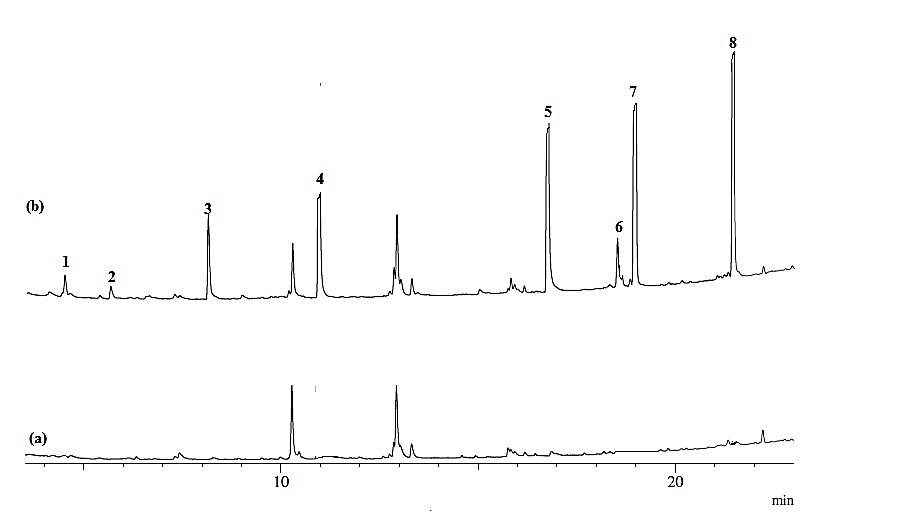
**

**Figure S6.** Chromatogram of mineral water; (**a**) unspiked; (**b**) spiked PAEs (50 µg·L^-1^). Peaks: (1) DMP; (2) DEP; (3) DPP; (4) DBP; (5) BBP; (6) DCP; (7) DEHP; (8) DNOP.
